# Genetic analysis of myeloid neoplasms with der(1;7)(q10;p10)

**DOI:** 10.1038/s41375-024-02494-2

**Published:** 2024-12-23

**Authors:** Rurika Okuda, Yotaro Ochi, Ryunosuke Saiki, Toshiyuki Yamanaka, Chikashi Terao, Tetsuichi Yoshizato, Masahiro M. Nakagawa, Lanying Zhao, Kazuma Ohyashiki, Nobuhiro Hiramoto, Masashi Sanada, Hiroshi Handa, Senji Kasahara, Yasushi Miyazaki, Nobuo Sezaki, Lee-Yung Shih, Wolfgang Kern, Nobuhiro Kanemura, Toshiyuki Kitano, Shinsaku Imashuku, Mitsumasa Watanabe, Maria Creignou, Kazuhisa Chonabayashi, Kensuke Usuki, Takayuki Ishikawa, Akihiko Gotoh, Yoshiko Atsuta, Yuichi Shiraishi, Kinuko Mitani, Shigeru Chiba, Akifumi Takaori-Kondo, Satoru Miyano, Yoichiro Kamatani, Torsten Haferlach, Eva Hellström-Lindberg, Koichi Matsuda, Yoshinori Yoshida, Hideki Makishima, Yasuhito Nannya, Seishi Ogawa

**Affiliations:** 1 Department of Pathology and Tumor Biology, Graduate School of Medicine, Kyoto University, Kyoto, Japan; 2 Laboratory for Statistical and Translational Genetics, RIKEN Center for Integrative Medical Sciences, Yokohama, Japan; 3 Clinical Research Center, Shizuoka General Hospital, Shizuoka, Japan; 4 The Department of Applied Genetics, The School of Pharmaceutical Sciences, University of Shizuoka, Shizuoka, Japan; 5 Department of Hematology, Tokyo Medical University, Tokyo, Japan; 6 Department of Hematology, Kobe City Medical Center General Hospital, Hyogo, Japan; 7 Department of Advanced Diagnosis, Clinical Research Center, NHO Nagoya Medical Center, Nagoya, Japan; 8 Department of Hematology, Graduate School of Medicine, Gunma University, Gunma, Japan; 9 Department of Hematology, Gifu Municipal Hospital, Gifu, Japan; 10 Japan Adult Leukemia Study Group, and Department of Hematology, Atomic Bomb Disease Institute, Nagasaki University, Nagasaki, Japan; 11 Department of Hematology, Chugoku Central Hospital, Hiroshima, Japan; 12 Division of Hematology-Oncology, Chang Gung Memorial Hospital–Linkou, Chang Gung University, Taoyuan, Taiwan; 13 MLL Munich Leukemia Laboratory, Munich, Germany; 14 Department of Hematology and Infectious Disease, Gifu University Graduate School of Medicine, Gifu, Japan; 15 Department of Hematology, Tazuke Kofukai, Medical Research Institute, Kitano Hospital, Osaka, Japan; 16 Department of Laboratory Medicine, Uji-Tokushukai Medical Center, Uji, Japan; 17 Department of Hematology, Hyogo Prefectural Amagasaki General Medical Center, Hyogo, Japan; 18 Department of Medicine Huddinge, Center for Hematology and Regenerative Medicine, Karolinska Institutet, Stockholm, Sweden; 19 Center for Clinical Cancer Studies, Phase 1 Unit, Karolinska University Hospital, Stockholm, Sweden; 20 Department of Cell Growth and Differentiation, Center for iPS Research and Application, Kyoto University, Kyoto, Japan; 21 Department of Hematology, Graduate School of Medicine, Kyoto University, Kyoto, Japan; 22 Department of Hematology, NTT Medical Center Tokyo, Tokyo, Japan; 23 The Japanese Data Center for Hematopoietic Cell Transplantation, Aichi, Japan; 24 Department of Registry Science for Transplant and Cellular Therapy, Aichi Medical University School of Medicine, Aichi, Japan; 25 Human Genome Center, Institute of Medical Science, The University of Tokyo, Tokyo, Japan; 26 Department of Hematology and Oncology, Dokkyo Medical University, Tochigi, Japan; 27 Department of Hematology, Institute of Medicine, University of Tsukuba, Ibaraki, Japan; 28 M&D Data Science Center, Tokyo Medical and Dental University, Tokyo, Japan; 29 Department of Computational Biology and Medical Sciences, Graduate Schools of Frontier Sciences, The University of Tokyo, Tokyo, Japan; 30 Department of Hematology, Karolinska University Hospital, Stockholm, Sweden; 31 Institute for the Advanced Study of Human Biology (WPI-ASHBi), Kyoto University, Kyoto, Japan

**Keywords:** Cancer genetics, Myelodysplastic syndrome

## To the Editor:

der(1;7)(q10;p10) is an unbalanced translocation recurrently found in a variety of myeloid neoplasms, where +1q and −7q are the common consequences [1–6]. Although it represents one of the most frequent chromosomal abnormalities in myeloid neoplasms among Asian populations, the molecular characteristics of der(1;7)(q10;p10)(+) myeloid neoplasms have not been fully elucidated. In this retrospective study, we enrolled 3,385 myeloid neoplasm cases to identify a total of 148 cases with der(1;7)(q10;p10) on the basis of conventional cytogenetics and/or sequencing-based copy number analysis [7] and investigated their clinical features and mutational profiles in comparison with those cases having −7/del(7q) (n = 376) and 1q trisomy (+1q) (n = 54) alone, using whole-exome sequencing (WES) and/or targeted-capture sequencing. The remaining 2,808 cases were collectively analyzed as “OTHER” cases (Supplementary Methods and Supplementary Table 1). Through these analyses, we demonstrated that der(1;7)(q10;p10)(+) myeloid neoplasms were characterized by unique clinical and mutational features and therefore, represented a distinct subset of myeloid neoplasms.

In accordance with previous reports [1, 4], there was an extreme male predominance in der(1;7)(q10;p10)(+) cases (87.8%)(*P* < 0.001) (Supplementary Table 1). der(1;7)(q10;p10) was significantly more prevalent in Asian (54/936) than German (4/944) myelodysplastic syndromes (MDS) cases (5.8% vs. 0.4%) (Data not shown). Compared to −7/del(7q) and +1q, der(1;7)(q10;p10) was more enriched (72.3%) for MDS than for acute myeloid leukemia (AML) (23.6%) and MDS/myeloproliferative neoplasm (MPN) (3.4%). MDS with excess blasts (MDS-EB) was less common in der(1;7)(q10;p10)(+) MDS cases than in −7/del(7q)(+) MDS patients, but more frequent compared to +1q cases. Secondary AML was more enriched in der(1;7)(q10;p10)(+) AML cases compared to non-der(1;7)(q10;p10) cases (Supplementary Fig. 1A). der(1;7)(q10;p10)(+) MDS cases had significantly higher hemoglobin levels, higher platelet counts, and lower blast counts than −7/del(7q) MDS cases (Supplementary Fig. 1B).

der(1;7)(q10;p10)(+) MDS, together with −7/del(7q)(+) MDS, had a significantly shorter overall survival and faster leukemic transformation than OTHER MDS cases, and in accordance with our previous report, [1] der(1;7)(q10;p10)(+) MDS cases tended to have a slightly better OS and slower leukemic progression than −7/del(7q) MDS cases (Supplementary Fig. 1C). By contrast, +1q MDS cases showed a trend for better OS. Compared to OTHER MDS cases, der(1;7)(q10;p10)(+) MDS cases tended to die without AML progression. Of note, infection-related deaths explained as many as 45% of non-leukemic deaths among der(1;7)(q10;p10)(+) MDS patients, whereas it explained only 13.9% and 10.8% for −7/del(7q) and OTHER MDS cases, respectively (Supplementary Fig. 1D). Interestingly, +1q MDS patients also showed a similar trend with 60% of cases dying from infection-related causes.

**Fig. 1:**
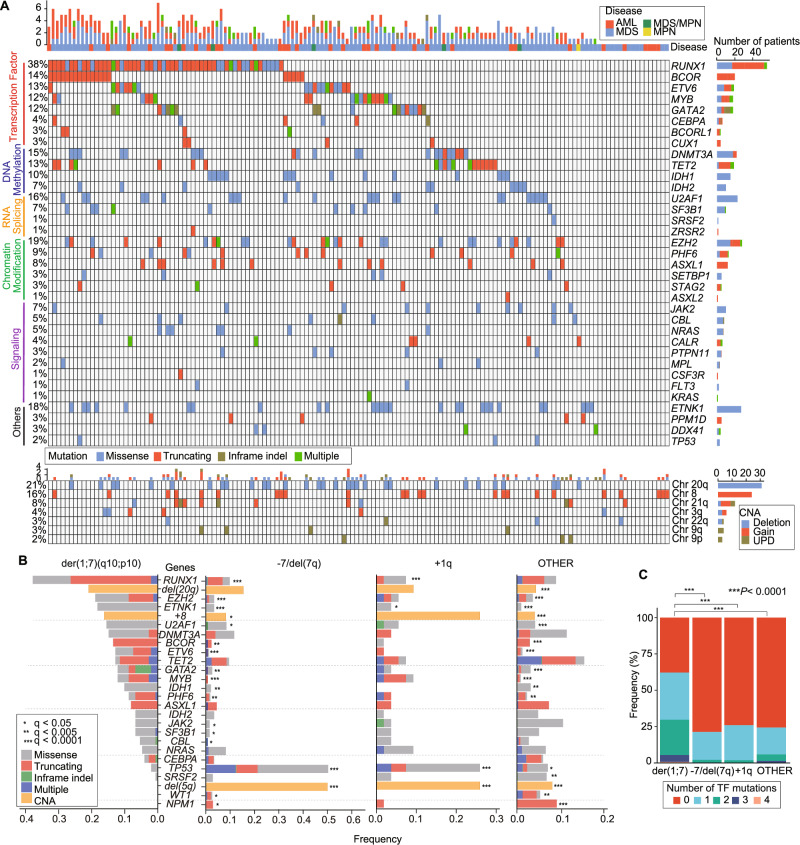
Genetic characteristics of der(1;7)(q10;p10)(+) myeloid neoplasms. **A** Landscape of gene mutations and copy number alterations (CNAs) in 148 der(1;7) (q10;p10)(+) myeloid neoplasm cases identified through targeted-capture sequencing. Number of genetic mutations per case shown as bar plots at top. Genetic mutation type and disease depicted as colors. Frequency of each mutation (>1%) and number of patients with the mutation are shown in left and right, respectively. Rows are ordered by functional categories of affected genes. **B** Frequency bar plot of targeted-sequencing gene mutation and CNA for der(1;7) (q10;p10)(+) vs. −7/del(7q) vs. +1q vs. others. Type of mutation depicted by differing colors. False discovery rate (q-values) shown by asterisks (*). Transcription factor genes in red. **C** Bar graph showing the frequency of the number of transcription factor mutations per case for each group (*P*-values calculated by Fisher’s Exact test). UPD uniparental disomy, TF transcription factor.

We next conducted targeted-capture sequencing for known driver genes and gene mutations identified through WES of 26 der(1;7)(q10;p10)(+) cases and revealed that 132 out of 148 der(1;7)(q10;p10)(+) cases (89.2%) had one or more gene mutations (Fig. 1A). The mean number of mutations was significantly higher in der(1;7)(q10;p10)(+) cases (2.8/sample) compared to other sub-groups (range, 1.7–1.9)(*P* < 0.001) (Supplementary Fig. 1E). Most frequently mutated genes in der(1;7)(q10;p10)(+) cases included *RUNX1, EZH2, ETNK1, U2AF1, DNMT3A, BCOR, ETV6, TET2, GATA2, MYB, IDH1, PHF6*, and *ASXL1*, the majority of which were more frequently mutated in der(1;7)(q10;p10)(+) cases than in other sub-groups (Fig. 1B and Supplementary Fig. 1F). Accounting for half of these genes, transcription factor genes, including *RUNX1, BCOR, ETV6, GATA2, MYB*, and *CEBPA*, represented major mutational targets in der(1;7)(q10;p10)(+) cases (Fig. 1C). Interestingly, we observed multiple mutations affecting single genes for some of these transcription factor genes, including *RUNX1*, *ETV6*, *MYB*, and *GATA2*, suggesting a strong selective pressure that favors these mutations within the der(1;7)(q10;p10)(+) populations (Fig. 1A). Overall, 62.2% of der(1;7)(q10;p10)(+) cases harbored at least one transcription factor mutation, a much higher frequency than der(1;7)(q10;p10)(−) cases (Fig. 1C). +8 and del(20q) were common in der(1;7)(q10;p10)(+) cases. By contrast, *TP53* mutations and del(5q) were rarely seen in der(1;7)(q10;p10)(+) cases, showing a sharp contrast to the very high frequencies of these alterations in −7/del(7q), +1q and OTHER cases (Supplementary Fig. 1F).

**Fig. 2:**
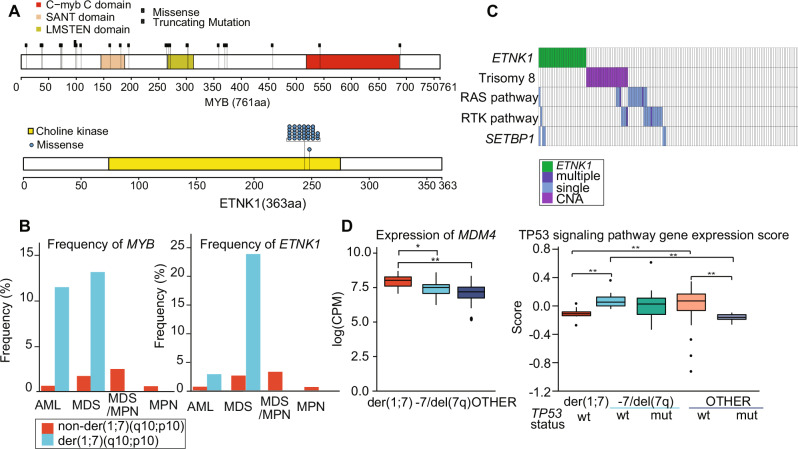
Characteristic mutations in der(1;7)(q10;p10)(+) myeloid neoplasms. **A** Lollipop plot of *MYB* and *ETNK1* gene mutations in der(1;7)(q10;p10) cases. Domains for each gene and type of mutations are depicted. **B** Frequency of *MYB* (left) and *ETNK1* mutations (right) in AML, MDS, MDS/MPN and MPN for der(1;7)(q10;p10)(+) and non-der(1;7)(q10;p10) cases. **C** Presence of indicated mutations according to *ETNK1* mutation status. RAS/RTK genes: *FLT3*, *JAK2*, *MPL*, *CALR*, *CSF3R*, *PTPN11*, *NF1*, *NRAS*, *KRAS*, and *CBL*. **D** Boxplot of *MDM4* gene expression (left) and expression level of p53 pathway genes (right) for each group. The median and 1st and 3rd quartiles are indicated, and whiskers extend to the furthest value within 1.5× the interquartile range. *P*-values calculated by Wilcoxon test. *P < 0.05, **P < 0.01.

Among frequent mutational targets of der(1;7)(q10;p10), two genes, *MYB* and *ETNK1*, were rarely mutated in OTHER and −7/del(7q) sub-groups, and therefore, are highly characteristic of der(1;7)(q10;p10)(+) cases. Interestingly, *MYB* mutations were also common in +1q cases **(**Fig. 1B). *MYB* is a proto-oncogene originally identified as the v-*Myb* oncogene within the genome of avian myeloblastosis virus and E26 virus [8]. Most variants were protein-truncating, and widely distributed along the entire coding sequence and, therefore, lead to a loss of function (Fig. 2A). *MYB* mutations were found in 13.1% of MDS and 11.4% of AML cases with der(1;7)(q10;p10)(+), but rarely detected in der(1;7)(q10;p10)(−) cases (Fig. 2B). There were no significant differences in complete blood counts between patients with and without *MYB* mutations (Supplementary Fig. 2A). Mutation profiles did not substantially differ between *MYB*-mutated (mut) and *MYB*-wild type (wt) cases. *MYB*-mut cases showed a shorter OS than *MYB*-wt cases, although not statistically significant. *ETNK1* was another gene uniquely mutated in der(1;7)(q10;p10)(+) cases. We found frequent *ETNK1* mutations in 18% of der(1;7)(q10;p10)(+) cases, which showed a prominent mutational hotspot affecting N244. Mutations were highly specific to MDS cases (24.3%) with only 2.9% in AML cases, which was comparable to der(1;7)(q10;p10)(−) MDS (2.6%) and MDS/MPN (3.3%) cases (Fig. 2A, B). As was the case with atypical chronic myeloid leukemia [9], *ETNK1* mutations significantly co-occurred with *SETBP1* mutations in der(1;7)(q10;p10)(+) myeloid neoplasms (*P* = 0.042) (Fig. 2C). By contrast, +8 tended to be mutually exclusive with *ETNK1* mutations (*P* = 0.0078) (Fig. 2C). Notably, *ETNK1*-mut der(1;7)(q10;p10)(+) cases presented with eosinophilia (mean: 6.2% vs. 14.8%) (*P* = 0.025) (Supplementary Fig. 2A). *ETNK1*-mutated MDS cases showed a poorer prognosis than *ETNK1*-wt cases.

Next, we examined gene expression profiles using RNA-sequencing of 10 der(1;7)(q10;p10)(+), 20 −7/del(7q), and 62 OTHER MDS patients. As expected, we confirmed the effect of allelic dosage: elevated and decreased expression across the genes on 1q and 7q, respectively, while the effect was highly variable for individual genes. For example, we noted a consistent reduction of gene expression of *EZH2* and *CUX1*, which are two major tumor suppressors and frequent targets of mutations and focal deletions on 7q in a variety of myeloid neoplasms, while elevated expression was observed for several oncogenes on 1q, such as *AKT3, BCL9, NCSTN, LAMC1, MDM4*, and *RIT1* (Supplementary Fig. 2B). Thus, deregulation of these tumor suppressors and oncogenes could explain the pathogenesis of der(1;7)(q10;p10)(+) MDS. Of particular interest among these is *MDM4* because MDM4 is a negative regulator of p53 functions and the lack of *TP53* mutation was another unique feature in der(1;7)(q10;p10)(+) cases. Elevated *MDM4* expression caused by 1q gain has been implicated in the paucity of *TP53* mutations in +1q cases in other tumors [10–12]. In line with these reports, down-regulated expression of p53 signaling pathway genes observed in *TP53*-mutated MDS with and without −7/del(7q), was also demonstrated in der(1;7)(q10;p10)(+) cases (Fig. 2D). *TP53* mutations were also frequent in cases with +1q alone, however, *TP53*-mutations tended to show a larger mutant cell fraction than that of +1q, suggesting that the +1q clone evolved from within the pre-existing *TP53*-mutant clone (Data not shown), implying a distinct pathogenesis between der(1;7)(q10;p10) and +1q alone.

Finally, to investigate the evolution of der(1;7)(q10;p10)(+) clones, we inferred the order of acquisition of genetic alterations by evaluating their variant allele frequency. der(1;7)(q10;p10), together with del(20q) and mutations in *DNMT3A* and *ETNK1*, represented the major clone in most cases, suggesting their early origin during clonal evolution. By contrast, characteristic transcription factor gene mutations in der(1;7)(q10;p10), such as *ETV6*, *GATA2*, and *MYB*, were relatively late events (Supplementary Fig. 2C, D). Furthermore, der(1;7)(q10;p10) was identified as clonal mosaicism in healthy individuals before they developed AML/MDS. When we surveyed CNAs among 179,417 healthy individuals from the BioBank Japan based on SNP array data [13, 14], we found 29 individuals harboring concomitant gain of 1q and loss of 7q suggestive of der(1;7)(q10;p10), of which four later died of AML or MDS (Supplementary Fig. 2E). Another interesting finding was the identification of *ETNK1* hotspot mutations in only individuals with der(1;7)(q10;p10) when we analyzed mutations by duplex-sequencing in 64 individuals including six with der(1;7)(q10;p10) (Supplementary Fig. 2F). Droplet Digital PCR (ddPCR) of 146 BBJ samples also identified these hotspot mutations in only those with der(1;7)(q10;p10) (Data not shown). Given the strong and unique association between *ETNK1* mutations and der(1;7)(q10;p10), *ETNK1* mutations may be acquired during early evolution of der(1;7)(q10;p10)(+) myeloid neoplasms as clonal hematopoiesis.

In conclusion, der(1;7)(q10;p10)(+) myeloid neoplasms are characterized by a number of unique genetic features and distinct hematological profiles and are therefore considered to represent a distinct entity of myeloid neoplasms, as supported by the recent findings from the IWG cohort [15]. Identification of this subset of myeloid neoplasms may allow for better prognostication and treatment for these patients. An elevated MDM4 expression associated with 1q gain indicates a possible role of MDM4 inhibitor for these neoplasms which needs to be tested in the clinical setting in the future.

## Supplementary information

Supplementary materials and methods, and figures

## Data Availability

Datasets of WES and RNA-seq data are available in the European Genome-phenome Archive database (Accession ID: EGAS50000000704 and EGAS50000000705).
