# Supplementary material for: Genetic analysis of myeloid neoplasms with der(1;7)(q10;p10)
Source: Leukemia. 2024 Dec 23;39(3):760–4. doi: 10.1038/s41375-024-02494-2 (PMC11879841; doi:10.1038/s41375-024-02494-2)
Supplement: Supplementary file 1 — Supplementary materials and methods, and figures [file 41375_2024_2494_MOESM1_ESM.pdf]

Supplementary Methods

Patient samples

We enrolled 148 der(1;7)(q10;p10) harboring myeloid neoplasm cases and 3,238 non-der(1;7)(q10;p10) myeloid neoplasm cases from collaborating institutes in this study (Supplementary Table 1). An additional 944 MDS cases from the German Munich Leukemia Laboratory was also included for the inter-racial comparison[1]. Whole bone marrow (WBM) samples, peripheral blood (PB) samples, and oral mucosa swabs were collected along with written informed consent in accordance with the Declaration of Helsinki and appropriate Ethics Committee approvals from patients from the following institutions: Kyoto University, Japan Marrow Donor Program, Tokyo Medical University, Kobe City Medical Center General Hospital, Gunma University, Gifu Municipal Hospital, Japan Adult Leukemia Study Group, Chang Gung University, Chugoku Central Hospital, University of Tsukuba, Gifu University Graduate School of Medicine, Kitano Hospital, Uji-Tokushukai Medical Center, Hyogo Prefectural Amagasaki General Medical Center, Dokkyo University, Karolinska Institute, NTT Medical Center Tokyo, Tokyo Metropolitan Cancer and Infectious Diseases Center Komagome Hospital, National Hospital Organization Nagoya Medical Center, Kitano Hospital.

Determination of der(1;7)(q10;p10) cases were conducted by utilizing sequenced-based copy number analysis of targeted-capture sequencing[2].

For an inter-racial incidence comparison, only Asian cases sequenced in consecutive cases were included to remove potential selection bias.

Whole-exome sequencing

For whole-exome sequencing, SureSelect Human All Exon v6 kits (Agilent Technologies) or xGen™ Exome Hyb Panel v2 were used to prepare 50ng of above patient DNA. Using Brrows-Wheeler Aligner, the resulting sequencing reads were aligned to the human genome reference (GRCh37/hg19)[3]. Mutations were called using GenomonPipeline[4], adopting parameters used in previous studies[2].

Targeted-capture sequencing

For targeted-capture sequencing, 200ng of above patient DNA were used for hybridization-based capture via SureSelect custom kit (Agilent Technologies) and sequenced on HiSeq 2500, NovaSeq6000 (Illumina) or MGI sequencer (MGI). Using Brrows-Wheeler Aligner, the resulting sequencing reads were aligned to

the human genome reference (GRCh37/hg19)[3]. Mutations were called using GenomonPipeline[4], adopting parameters used in previous studies[2].

RNA-sequencing

For RNA-sequencing, RNA was extracted from ten der(1;7)(q10;p10)(+), 20 -7/del(7q), and 62 OTHER MDS patient BM samples, and CD34+ cell selection was conducted using CD34 MicroBead Kit (Miltenyi Biotec) according to the manufacturer's instructions. Libraries were prepared using NEBNext Ultra II RNA Library Prep Kit for Illumina (New England BioLabs) and sequenced with NovaSeq 6000 instrument (Illumina). The reads were mapped to a reference genome (hg19) using STAR(v2.5.3)[5], counted using featureCounts[6], and differentially expressed genes were identified by edgeR[7] at FDR<0.05. 'Score' for TP53 pathways were calculated using the $\log_2$(CPM) values for genes listed in the 'HALLMARK_P53_PATHWAY' from the Molecular Signatures Database (MSigDB)[8]: and using the R function 'scale' to scale the $\log_2$(CPM) values.

Duplex-sequencing and Digital droplet PCR (ddPCR)

Sequencing was conducted upon 54 samples from healthy individuals in the BBJ cohort using xGen Prism prep kit (IDT), following the manufacturer's instructions. For target enrichment, we designed a custom IDT xGen bait to cover 29 genes commonly mutated in der(1;7)(q10;p10)(+) myeloid neoplasms and genes relating to clonal hematopoiesis (CH). Sequencing was performed on DNBSEQ-G400RS (MGI Tech) in 100-150 bp paired-end mode, according to the manufacturer's instructions. Original sequence reads were mapped by BWA (https://github.com/lh3/bwa), followed by conversion into Bam via Picard (https://broadinstitute.github.io/picard/). Duplex consensus sequences were extracted and processed according to the manufacturer's instructions. Sequences were collapsed into families by fgbio CallDuplexConsensusReads and mutations were called using Genomon Pipeline v2.6.2[4]. Copy number alterations (CNAs) on chromosomes 1q, 2, 7q, 8, and 20q were evaluated by B-allele frequencies (BAF) of heterozygous SNPs calculated by GenomonMutationFilter (https://github.com/Genomon-Project/GenomonMutationFilter) and normalization with the average BAF of SNPs on chr2, which were not affected by CNAs in analyzed cases. For ddPCR, predesigned *ETNK1* p.N244S c.731A>G probes (Catalog # 10049047) were purchased from Bio-Rad. Droplet generation was conducted on the QX200 Droplet Generator for each DNA/enzyme mix (50ng of gDNA + ddPCR Supermix for Probes (no dUTP), Bio-Rad + HaeIII). PCR annealing temperatures were set at 55°C. Droplets were analyzed using QX200 system and QuantaSoft1.7 (Bio-Rad) according to the manufacturer's instructions.

Definitions for gene groups

Genes were categorized as follows according to the gene function[9].

Transcription factor: *RUNX1*, *BCOR*, *ETV6*, *GATA2*, *MYB*, *CEBPA*, *BCORL1*, and *CUX1*.

RAS/RTK genes: *FLT3*, *JAK2*, *MPL*, *CALR*, *CSF3R*, *PTPN11*, *NF1*, *NRAS*, *KRAS*, and *CBL*.

Clonal hematopoiesis (CH)-related genes[10]: *DNMT3A*, *TET2*, *ASXL1*, *SF3B1*, *PPM1D*, *JAK2*, and *CBL*.

Data Sharing Statement

Datasets of WES and RNA-seq data are available in the European Genome-phenome Archive database (Accession ID: EGAS50000000704 and EGAS50000000705).

Supplementary References

1. Haferlach, T. *et al.* Landscape of genetic lesions in 944 patients with myelodysplastic syndromes. *Leukemia* **28**, 241–247 (2014).
2. Yoshizato, T. *et al.* Genetic abnormalities in myelodysplasia and secondary acute myeloid leukemia: impact on outcome of stem cell transplantation. *Blood* **129**, 2347–2358 (2017).
3. Li, H. & Durbin, R. Fast and accurate short read alignment with Burrows-Wheeler transform. *Bioinformatics* **25**, 1754–1760 (2009).
4. Shiraishi, Y. *et al.* An empirical Bayesian framework for somatic mutation detection from cancer genome sequencing data. *Nucleic Acids Res.* **41**, (2013).
5. Dobin, A. *et al.* STAR: Ultrafast universal RNA-seq aligner. *Bioinformatics* **29**, 15–21 (2013).
6. Liao, Y., Smyth, G. K. & Shi, W. FeatureCounts: An efficient general purpose program for assigning sequence reads to genomic features. *Bioinformatics* **30**, 923–930 (2014).
7. Robinson, M. D., McCarthy, D. J. & Smyth, G. K. edgeR: A Bioconductor package for differential expression analysis of digital gene expression data. *Bioinformatics* **26**, 139–140 (2009).
8. Subramanian, A. *et al. Gene Set Enrichment Analysis: A Knowledge-Based Approach for Interpreting Genome-Wide Expression Profiles*. (2005).
9. Ogawa, S. Genetics of MDS. *Blood* **133**, 1049–1059 (2019).
10. Saiki, R. *et al.* Combined landscape of single-nucleotide variants and copy number alterations in clonal hematopoiesis. *Nat. Med.* **27**, 1239–1249 (2021).

Supplementary Table 1

Supplementary Table 1. Summary of clinical information of cohort analyzed.

	der(1;7)(q10;p10)			-7/del(7q)				+1q				OTHER			
							P-value				*P*-value				*P*-value
**No. of patients**	148.0			376.0				54.0				2808.0			
**Age at diagnosis (median (range)) - yrs.**	63.8	16-90		64.9	17-90		0.42	61.4	17-87		0.264	59.7	10-94		0.006
**Gender - no. (%)**															
Male	130.0	87.84		269.0	71.54		< 0.001	36.0	66.67		0.001	1695.0	60.36		< 0.001
Female	18.0	12.16		107.0	28.46			18.0	33.33			1113.0	39.64		
**WHO classification at sampling - no. (%)**															
**MDS**	**(n=107)**	**% Total**	**% Disease**	**(n=163)**	**% Total**	**% Disease**		**(n=29)**	**% Total**	**% Disease**		**(n=807)**	**% Total**	**% Disease**	
isolated del(5q)	0.0	0.00	0.00	0.0	0.00	0.00	na	0.0	0.00	0.00	na	14.0	0.50	1.73	0.392
MDS_SLD	17.0	11.49	15.89	4.0	1.06	2.45	< 0.001	4.0	7.41	13.79	1	112.0	3.99	13.88	0.556
MDS_MLD	34.0	22.97	31.78	31.0	8.24	19.02	0.02	18.0	33.33	62.07	0.005	217.0	7.73	26.89	0.300
MDS_RS	0.0	0.00	0.00	1.0	0.27	0.61	1	0.0	0.00	0.00	na	4.0	0.14	0.50	1
MDS_RS_SLD	0.0	0.00	0.00	2.0	0.53	1.23	0.52	0.0	0.00	0.00	na	32.0	1.14	3.97	0.025
MDS_RS_MLD	0.0	0.00	0.00	4.0	1.06	2.45	0.155	1.0	1.85	3.45	0.213	24.0	0.85	2.97	0.100
MDS_EB	43.0	29.05	40.19	108.0	28.72	66.26	< 0.001	5.0	9.26	17.24	0.028	323.0	11.50	40.02	1
MDS_U	8.0	5.41	7.48	6.0	1.60	3.68	0.261	0.0	0.00	0.00	0.202	32.0	1.14	3.97	0.125
unknown	5.0	3.38	4.67	7.0	1.86	4.29		1.0	1.85	3.45		49.0	1.75	6.07	
**AML**	**(n=35)**	**% Total**	**% Disease**	**(n=181)**	**% Total**	**% Disease**		**(n=18)**	**% Total**	**% Disease**		**(n=1232)**	**% Total**	**% Disease**	
AML_recurrent_genetic_abnormality	0.0	0.00	0.00	16.0	4.26	8.84	0.081	2.0	3.70	11.11	0.111	281.0	10.01	22.81	< 0.001
AML_MRC	28.0	18.92	80.00	162.0	43.09	89.50	0.151	10.0	18.52	55.56	0.106	430.0	15.31	34.90	< 0.001
tAML	3.0	2.03	8.57	3.0	0.80	1.66	0.055	0.0	0.00	0.00	0.543	12.0	0.43	0.97	0.007
AML_NOS	2.0	1.35	5.71	0.0	0.00	0.00	0.026	4.0	7.41	22.22	0.164	355.0	12.64	28.81	0.002
unknown	2.0	1.35	5.71	0.0	0.00	0.00		2.0	3.70	11.11		154.0	5.48	12.50	
**MDS/MPN**	**(n=5)**	**% Total**	**% Disease**	**(n=13)**	**% Total**	**% Disease**		**(n=2)**	**% Total**	**% Disease**		**(n=107)**	**% Total**	**% Disease**	
MDS/MPN_atypical CML	0.0	0.00	0.00	2.0	0.53	15.38	1	1.0	1.85	50.00	0.268	8.0	0.28	7.48	1
MDS/MPN_CMML	3.0	2.03	60.00	6.0	1.60	46.15	0.624	1.0	1.85	50.00	0.846	65.0	2.31	60.75	0.974
MDS/MPN_RS_T	0.0	0.00	0.00	0.0	0.00	0.00	na	0.0	0.00	0.00	na	4.0	0.14	3.74	1
MDS/MPN_U	2.0	1.35	40.00	5.0	1.33	38.46	1	0.0	0.00	0.00	na	29.0	1.03	27.10	0.616
unknown	0.0	0.00	0.00	0.0	0.00	0.00	na	0.0	0.00	0.00	na	1.0	0.04	0.93	
**MPN**	**(n=1)**	**% Total**	**% Disease**	**(n=19)**	**% Total**	**% Disease**		**(n=5)**	**% Total**	**% Disease**		**(n=662)**	**% Total**	**% Disease**	
MPN_ET	0.0	0.00	0.00	7.0	1.86	36.84	1	3.0	5.56	60.00	1	216.0	7.69	32.63	1
MPN_PV	0.0	0.00	0.00	1.0	0.27	5.26	1	0.0	0.00	0.00	na	114.0	4.06	17.22	1
MPN_PMF	1.0	0.68	100.00	0.0	0.00	0.00	0.05	1.0	1.85	20.00	0.333	51.0	1.82	7.70	0.078
MPN_CNL	0.0	0.00	0.00	0.0	0.00	0.00	na	0.0	0.00	0.00	na	7.0	0.25	1.06	1
MPN_CML_BCR/ABL	0.0	0.00	0.00	10.0	2.66	52.63	1	1.0	1.85	20.00	1	240.0	8.55	36.25	1
MPN_CEL_NOS	0.0	0.00	0.00	0.0	0.00	0.00	na	0.0	0.00	0.00	na	1.0	0.04	0.15	1
MPN_NOS	0.0	0.00	0.00	0.0	0.00	0.00	na	0.0	0.00	0.00	na	4.0	0.14	0.60	1
MPN_U	0.0	0.00	0.00	1.0	0.27	5.26	1	0.0	0.00	0.00	na	21.0	0.75	3.17	1
unknown	0.0	0.00	0.00	0.0	0.00	0.00	na	0.0	0.00	0.00		8.0	0.28	1.21	
**Peripheral Blood [MDS]**	**(n=106)**			**(n=163)**				**(n=29)**				**(n=807)**			
WBC (mean (range)) /uL	3238.6	600-17800		4362.7	700-44900		0.101	3148.8	200-9290		0.871	4470.4	230-84600		0.060
HB (mean (range)) g/dL	9.0	4.7-14.3		8.2	4.1-23.8		0.027	8.6	4.2-12.6		0.359	9.2	2.7-18.4		0.365
Plt (mean (range)) x10^4/L	19.8	0.5-124.7		9.6	0.1-92.8		< 0.001	7.2	0.09-22.9		0.015	12.8	0.1-177.8		< 0.001
**Bone Marrow [MDS]**	**(n=106)**			**(n=163)**				**(n=29)**				**(n=807)**			
Blast (mean (range)) %	4.8	0-19.4		7.4	0-19.5		< 0.001	2.6	0-13.2		0.017	5.0	0-19.6		0.693
**Peripheral Blood [AML]**	**(n=35)**			**(n=181)**				**(n=18)**				**(n=1232)**			
WBC (mean (range)) /uL	3787.2	600-33100		16069.8	50-222880		0.045	21582.7	670-120500		0.024	36615.6	230-676000		0.013
HB (mean (range)) g/dL	7.7	4-11.5		8.0	4-26.3		0.529	10.1	4.8-15.3		0.005	9.1	3.2-79		0.076
Plt (mean (range)) x10^4/L	4.1	1-14.8		7.9	0.3-91.3		0.065	5.4	0.2-17.3		0.371	9.9	0.2-505		0.192
**Bone Marrow [AML]**	**(n=35)**			**(n=181)**				**(n=18)**				**(n=1232)**			
Blast (mean (range)) %	34.2	20.7-73		43.6	20-98.4		0.055	49.2	22.3-90		0.19	39.4	20-100		0.034

Supplementary Table 1. Summary of clinical information of cohort analyzed.

Supplementary Fig. 1

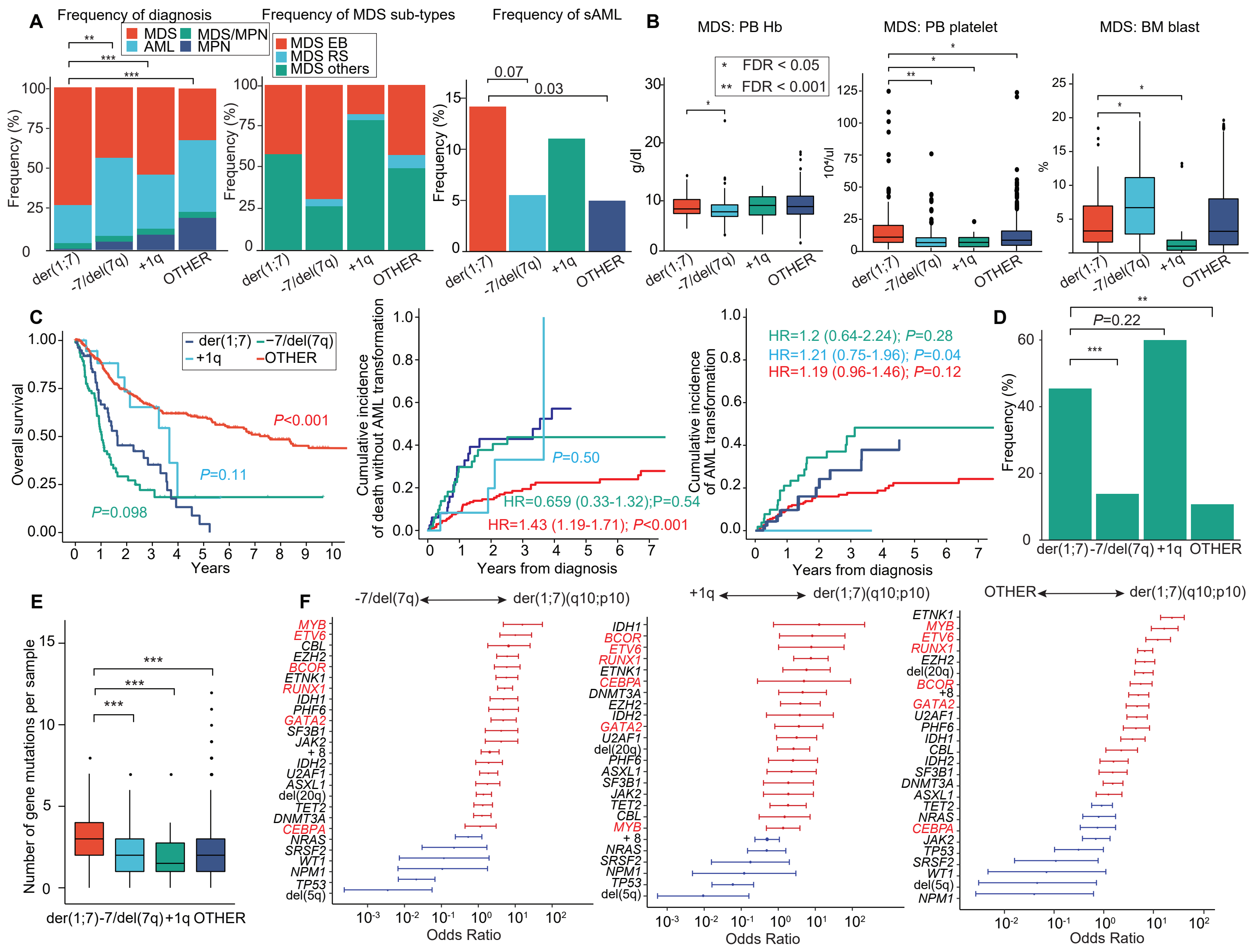

Supplementary Fig. 1. Clinical and genetic characteristics of der(1;7)(q10;p10)(+) myeloid neoplasms.
(A) Myeloid neoplasm disease distribution (left), MDS diagnosis distribution (middle), and Frequency of secondary AML (right) for der(1;7)(q10;p10)(+), -7/del(7q), +1q, and OTHER (Fisher's Exact Test). **P<0.01, ***P<0.001 (B) Box plot of peripheral blood hemoglobin (Hb) count, platelet count and bone marrow blast count of der(1;7)(q10;p10)(+), -7/del(7q), +1q and OTHER. Boxplot depicts median, 1st and 3rd quartile. False discovery rate (FDR) calculated by Benjamin-Hochberg procedure. (C) Kaplan-Meier curves for overall survival (left), cumulative incidence of death without AML transformation (middle), and cumulative incidence of AML transformation (right) of der(1;7)(q10;p10)(+), -7/del(7q), +1q, and OTHER. Number at risk shown below the curve for Kaplan-Meier curve. Hazard ratios (HR) and P-values relative to der(1;7)(q10;p10)(+) for cumulative incidence curves. (D) Bar graph showing the fraction of infection-related deaths in der(1;7)(q10;p10)(+) vs. -7/del(7q) vs. +1q vs. OTHER MDS cases (Fisher's Exact Test). **P<0.01, ***P<0.001 (E) Boxplot for number of additional genetic events per case. The median and 1st and 3rd quartiles are indicated, and whiskers extend to the furthest value within 1.5× the interquartile range. P-values calculated by Wilcoxon test. ***P<0.001 (F) Forest plot of mutation frequency odds ratio. Each sub-group vs der(1;7)(q10;p10)(+). Error bars indicate 95% confidence interval. Transcription factor genes in red.

Supplementary Fig. 2

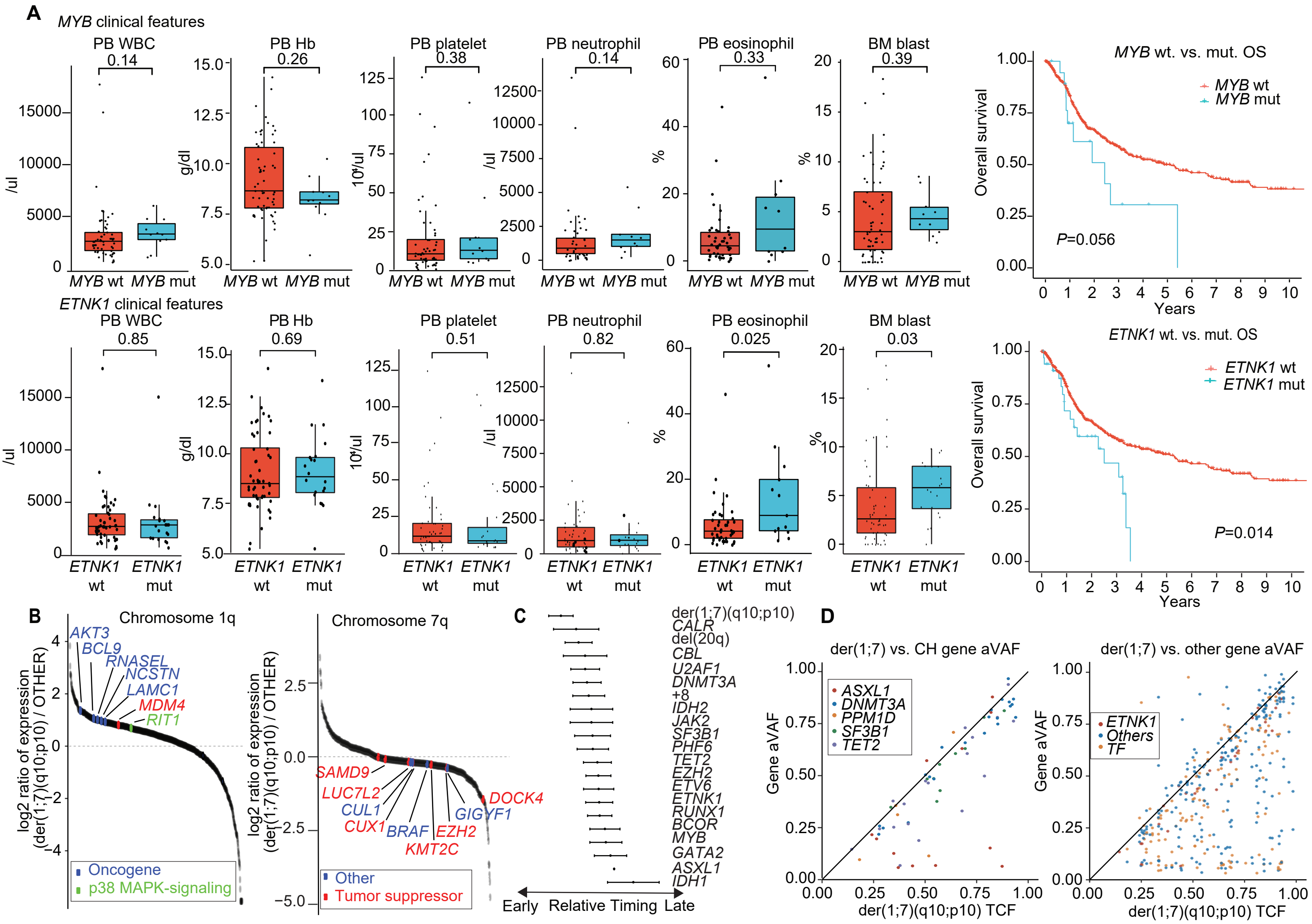

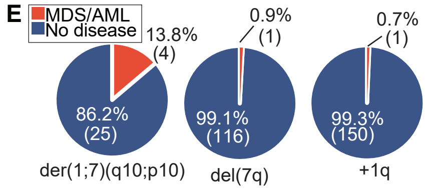

F

sub-group	*ETNK1* mutations	total	*P*-value
der(1;7)(q10;p10)	2	6	-
+1q	0	8	0.16
del(7q)	0	10	0.13
OTHER	0	30	0.024
sum of non-der(1;7) BBJ	0	48	0.011

Supplementary Fig. 2. Characteristic gene mutations of der(1;7)(q10;p10).
(A) Boxplot of peripheral blood white blood cell (WBC) count, hemoglobin (Hb) level, platelet count, neutrophil count, eosinophil level, and bone marrow blast percentage count (Wilcoxon test P-values) and Kaplan-Meier curve for overall survival of all MDS cases comparing wild type (wt)(red) vs. mutated (mut)(blue) cases for *MYB* (above) and *ETNK1* (below). (B) Dot plot depicting log ratio of expression of genes comparing der(1;7)(q10;p10) with OTHER group. Genes on chromosomes 1q and 7q are depicted in (left) and (right), respectively. Key genes colored as follows: Oncogene (blue) and p38 MAPK-signaling (green) on (left); Tumor suppressor (red) and Other known genes (blue) on (right).(C) Bradley-Terry analysis result for gene mutations and CNAs involved in at least five pairwise precedence. The relative temporal order shown with respect to *ASXL1*. Standard errors shown as horizontal bars. (D) Scatter plot of tumor cell fraction (TCF) of der(1;7)(q10;p10) (x-axis) and adjusted variable allele frequency (aVAF) of other mutations (y-axis). y-axis represents aVAF of CH related genes (*DNMT3A*, *TET2*, *ASXL1*, *SF3B1*, and *PPM1D*) in (right), and transcription factor genes (*RUNX1*, *BCOR*, *ETV6*, *GATA2*, *MYB*, and *PHF6*) (TF), *ETNK1*, and other genes on the left. Each dot represents each mutation. (E) Pie chart showing the development of MDS or AML for der(1;7)(q10;p10), del(7q) and +1q harboring individuals. (F) Table showing number of *ETNK1* mutations found in each sub-group through duplex-sequencing. (Fisher's Exact Test)
